# A Versatile Surface Bioengineering Strategy Based on Mussel-Inspired and Bioclickable Peptide Mimic

**DOI:** 10.34133/2020/7236946

**Published:** 2020-06-25

**Authors:** Yu Xiao, Wenxuan Wang, Xiaohua Tian, Xing Tan, Tong Yang, Peng Gao, Kaiqing Xiong, Qiufen Tu, Miao Wang, Manfred F. Maitz, Nan Huang, Guoqing Pan, Zhilu Yang

**Affiliations:** ^1^Key Laboratory of Advanced Technologies of Materials, Ministry of Education, School of Materials Science and Engineering, Southwest Jiaotong University, Chengdu, Sichuan 610031, China; ^2^Institute for Advanced Materials, School of Materials Science and Engineering, Jiangsu University, Zhenjiang, Jiangsu 212013, China; ^3^Max Bergmann Center of Biomaterials, Leibniz Institute of Polymer Research Dresden, Hohe Strasse 6, 01069 Dresden, Germany

## Abstract

In this work, we present a versatile surface engineering strategy by the combination of mussel adhesive peptide mimicking and bioorthogonal click chemistry. The main idea reflected in this work derived from a novel mussel-inspired peptide mimic with a bioclickable azide group (i.e., DOPA_4_-azide). Similar to the adhesion mechanism of the mussel foot protein (i.e., covalent/noncovalent comediated surface adhesion), the bioinspired and bioclickable peptide mimic DOPA_4_-azide enables stable binding on a broad range of materials, such as metallic, inorganic, and organic polymer substrates. In addition to the material universality, the azide residues of DOPA_4_-azide are also capable of a specific conjugation of dibenzylcyclooctyne- (DBCO-) modified bioactive ligands through bioorthogonal click reaction in a second step. To demonstrate the applicability of this strategy for diversified biofunctionalization, we bioorthogonally conjugated several typical bioactive molecules with DBCO functionalization on different substrates to fabricate functional surfaces which fulfil essential requirements of biomedically used implants. For instance, antibiofouling, antibacterial, and antithrombogenic properties could be easily applied to the relevant biomaterial surfaces, by grafting antifouling polymer, antibacterial peptide, and NO-generating catalyst, respectively. Overall, the novel surface bioengineering strategy has shown broad applicability for both the types of substrate materials and the expected biofunctionalities. Conceivably, the “clean” molecular modification of bioorthogonal chemistry and the universality of mussel-inspired surface adhesion may synergically provide a versatile surface bioengineering strategy for a wide range of biomedical materials.

## 1. Introduction

Advanced biomedical implants should have the abilities to actively integrate the surrounding tissue, communicate with surrounding cells, trigger cell responses, maintain tissue and organ functions, combat hostile microorganisms, etc. [1, 2]. In this regard, surface biofunctionalization represents one of the most straightforward ways to endow biomaterials with such “vitality” [3–5]. Physical adsorption or chemical conjugation is a typical method for surface modification with bioactive ligands, which enables inherently bioinert materials to modulate cell-material interactions, induce specific cell behaviors, and subsequently generate relevant biological effects [6–8].

Common physical means for surface biofunctionalization, such as surface layer-by-layer assembly [9] and Langmuir-Blodgett deposition [10], depend on noncovalent molecular bindings. These weak interactions inevitably result in biomolecular desorption and subsequently the lack of long-term activity. Although chemical conjugations show stronger molecular anchoring, current chemical means frequently still suffer from tedious reactions as well as complex surface treatment technologies [11, 12]. Moreover, these traditional methods for surface biofunctionalization (i.e., physical binding or chemical conjugation) are not equally applicable on a wide range of material surfaces but require specific adaptation. In this context, a novel surface engineering method, inspired by the marine mussel adhesion, was developed in 2007 [13]. The molecular mechanism of this method derived from mussel foot proteins (e.g., *Mytilus edulis* foot proteins, Mfps), in which the repetitive catechol residues of DOPA (3,4-dihydroxy-L-phenylalanine) can produce covalent and noncovalent comediated molecular adhesion [14]. A great deal of studies indicates that Mfps-mimics (e.g., polydopamine [15–17], DOPA-rich peptides [18, 19], and catecholic polymers [20, 21]) with catechol groups can adhere stably to virtually all kinds of substrates under wet conditions [22]. In addition, a second-step conjugation with bioactive molecules through amino- or thiol-mediated Michael addition allows a variety of biofunctionalizations. Undoubtedly, mussel-inspired molecular adhesion can provide a potentially universal strategy for surface bioengineering [23, 24].

Despite the simplicity and generality for diversified materials, current mussel-inspired surface strategies still are critically limited with respect to biomolecular modification. First, the second-step chemical conjugation through Michael addition or Schiff base potentially impedes the function of the biomolecule by consumption of essential amino and thiol groups [25]. Second, the Michael addition or Schiff base has only low specificity and efficiency, taking a toll on the reproducibility and controllability (e.g., heterogeneous molecular conjugation and random molecular orientation) [26]. Therefore, advanced modification technologies of current Mfps mimics are still demanded for improved surface bioengineering with easy operability, good controllability, and high reproducibility.

**Scheme 1:**
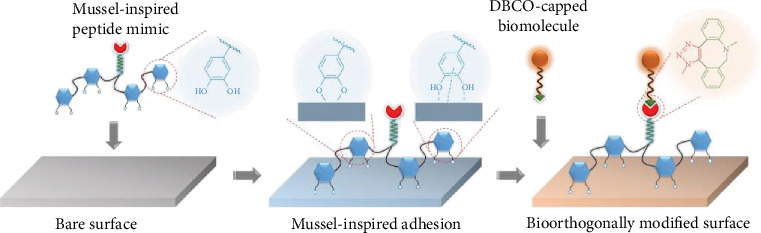
The molecular binding mechanisms of mussel-inspired peptide adhesion and bioorthogonal molecular conjugation for surface bioengineering.

Herein, we report an advanced surface bioengineering strategy by the combination of mussel-inspired molecular adhesion and bioorthogonal click chemistry (Scheme 1). In contrast to classical chemistry, bioorthogonal click reaction (e.g., the dibenzylcyclooctyne-azide (DBCO-azide) cycloaddition chemistry) shows advantages like specificity, rapidity, thoroughness, and biocompatibility [27, 28]. Thus, we considered designing an azide-bearing peptide with multiple catechol groups, mimicking the molecular properties of Mfps. Similar to the Mfps adhesion mechanism, the azide-bearing mussel adhesive peptide can stably bind onto a wide range of material surfaces via the covalent and noncovalent comediated molecular adhesion. Subsequently, the surface anchored azide groups enable a specific grafting of DBCO-modified bioactive ligands through DBCO-azide click reaction in a second step. Since DBCO modification is industrially mature and commercially available for biomolecules, we anticipate that the bioclickable mussel-inspired peptide might provide a flexible and more precise strategy for surface biofunctionalization.

As a proof of principle, we synthesized several typical DBCO-modified biomolecules with abilities to modulate cell-material interactions and induce specific biological effects. The basic and essential requirements of biomedical implants, such as antibiofouling [29, 30], antibacterial [31], and antithrombotic activity [32], were separately introduced onto different substrate materials corresponding to clinically applied medical devices. We demonstrated that the surface bioengineering strategy based on bioclickable and mussel adhesive peptide mimic had broad applicability in both the types of substrate materials and the intended functions. The “clean” molecular modification of bioorthogonal click chemistry and universal surface adhesion of mussel-inspired chemistry may synergically provide a versatile surface bioengineering strategy for a wide range of biomedical materials.

## 2. Results and Discussion

### 2.1. Bioclickable, Mussel Adhesive Peptide Mimic

**Figure 1:**
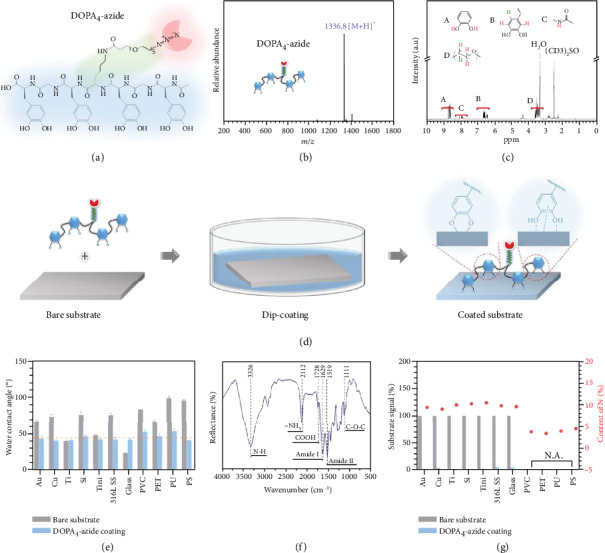
(a) Structural formula of the bioclickable mussel-inspired peptide mimic DOPA_4_-azide with four catechol groups and one azide group. (b) ESI mass spectrum of DOPA_4_-azide. (c) ^1^H NMR spectrum of DOPA_4_-azide. (d) Schematic illustration of the mussel-inspired peptide mimic for surface modification via catechol-mediated molecular adhesion. (e) The changes of surface wettability on different substrates after DOPA_4_-azide coating. TiNi: Ti-Ni alloy; 316L SS: 316 low carbon stainless steel; PVC: polyvinyl chloride; PET: polyethylene terephthalate; PU: polyurethane; PS: polystyrene. (f) GATR-FTIR spectrum of the DOPA_4_-azide coating on Au substrate. (g) The changes of substrate signal and N elemental content after DOPA_4_-azide coating.

The azide-bearing mussel adhesive peptide mimic was designed based on published sequences and prepared by standard Fmoc-mediated solid-phase peptide synthesis [33–35]. To mimic the multiple catechol structure in Mfps [36], acetonide-protected DOPA (i.e., Fmoc-DOPA (acetone)-OH) was programmatically linked into the main chain of peptide with one glycine (G) or lysine (K) spacer, leading to a mussel-inspired peptide with tetravalent DOPA sequence (i.e., DOPA-G-DOPA-K-DOPA-G-DOPA). Glycine and lysine act as the spacers to improve molecular twisting and facilitate the Mfps-like molecular adhesion. The gamma amino group of lysine was linked with an azide-terminated poly(ethylene glycol) (PEG), finally obtaining a clickable mussel-inspired peptide mimic DOPA-G-DOPA-K(PEG-azide)-DOPA-G-DOPA (i.e., DOPA_4_-azide, Figure 1(a)). The peptide was then cleaved from resin and purified through high-performance liquid chromatography (HPLC) (purity: 98.1%). Electrospray ionization mass spectrometry (ESI-MS) and nuclear magnetic resonance (NMR) spectroscopy were used to confirm the success of molecular synthesis. As shown in Figure 1(b), the monoisotopic mass [M+H]^+^ of DOPA_4_-azide was found at 1336.8 Da, which is in line with its theoretical molecular weight (1335.6 Da) of the chemical structure. The spectrum of ^1^H NMR indicated the presence of several diagnostic peaks, including the catecholic and aromatic hydrogens of DOPA, the amide hydrogens, and the hydrogens of ethylene glycol repeating units (Figure 1(c)). These results jointly confirmed the successful synthesis of the bioclickable mussel adhesive peptide mimic.

### 2.2. Diversity of Surface Adhesion

We then investigated the applicability of DOPA_4_-azide for surface modification of diversified materials. The materials are widely used for biomedical implants and are commonly very demanding for surface biofunctionalization, such as metals, inorganic materials, and organic polymers. The coating process was carried out by incubating the clean substrates in PBS solutions containing 0.1 mg·mL^−1^ of DOPA_4_-azide for 1 h (Figure 1(d)). As shown in Figure 1(e), the substrates exhibited significant changes in surface wettability. All the DOPA_4_-azide-coated surfaces showed water contact angles with a rough regression value around 45° (dashed line), attributed to the high hydrophilicity of the PEG chain in DOPA_4_-azide. The typical chemical composition of the DOPA_4_-azide-coating on Au substrate was then characterized by grazing incidence attenuated total reflection Fourier transform infrared (GATR-FTIR) spectroscopy (Figure 1(f)). Besides the characteristic peaks of peptide bonds, such as the carboxylic acid (1728 cm^−1^, C=O stretching), the amide I band (1629 cm^−1^, C=O stretching), and amide II band (1519 cm^−1^, N-H stretching), a characteristic peak of azide at 2112 cm^−1^ was also found. X-ray photoelectron spectroscopy (XPS) analysis was further performed to examine the surface elemental compositions (Figure 1(g) and Figure S1). After DOPA_4_-azide-coating, a significant N1s signal and efficient shielding of the substrate signal were observed for the metallic and inorganic materials. The signals of polymeric materials (e.g., the carbon-based PVC, PET, PU, and PS) were hard to be distinguished, but there was a remarkably enhanced N1s signal after coating. These results demonstrated the versatility of DOPA_4_-azide for surface modification of different classes of materials.

### 2.3. Antibiofouling Surface

**Figure 2:**
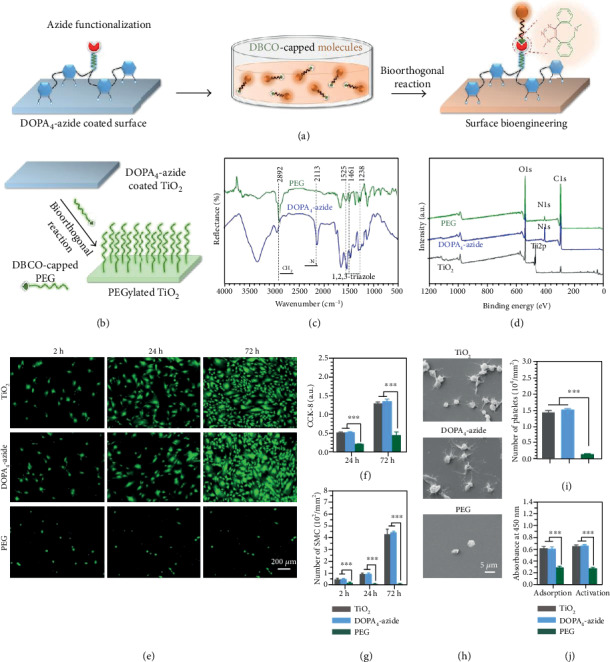
(a) Schematic illustration of the DOPA_4_-azide-coated substrate for second-step surface biomodification through bioorthogonal DBCO-azide click reaction. (b) Bioorthogonal PEGylation on the TiO_2_ surface using DBCO-PEG. (c) GATR-FTIR spectra of the DOPA_4_-azide-coated and PEGylated surfaces. (d) XPS analysis of the TiO_2_ surfaces at each step of surface treatments. (e) SMC adhesion at 2, 24, and 72 h. (f, g) SMC proliferation by the CCK-8 assay and cell counting. (h) Scanning electron microscope (SEM) images of adherent blood platelets. (i) Average numbers of adherent blood platelets. (j) Fibrinogen absorption and activation. Statistically significant differences are indicated by ^∗∗∗^*p* < 0.001.

Since the bioinspired and bioclickable peptide mimic could be coated on various substrates via mussel-inspired adhesion and lead to azide-functionalized surfaces, a second-step bioorthogonal grafting process in solution with DBCO-capped molecules was further investigated (Figure 2(a)). DBCO is a bulky cycloalkyne which reacts specifically with azides through copper-free (i.e., catalyst-free), strain-promoted azide-alkyne cycloaddition (SPAAC) [27]. Owing to the high specificity, efficient kinetics, and high compatibility in biosystems, bioorthogonal DBCO-azide click chemistry has been widely used for molecular conjugations both *in vitro* and *in vivo* [28]. As a proof of concept, we first employed a DBCO-terminated PEG molecule (Mw = 5000) to form a PEGylated antibiofouling surface. Biofouling, adsorption of biomolecules and cells and subsequent loss of function, is an ongoing problem in the field of biochips and biosensors in contact with biological fluids *in vitro* or *in vivo* [37, 38]. Herein, the bioorthogonally PEGylated antifouling surface was fabricated on a TiO_2_-deposited quartz slide (Figure 2(b)), because surface TiO_2_ deposition is widely used on biomedical devices (e.g., vascular stents). GATR-FTIR analysis was used to confirm the success of PEGylation. As shown in Figure 2(c), the azide peak in FTIR spectra disappeared, accompanied by the appearance of a group of triazole peaks after bioorthogonal PEGylation, indicating the efficient bioorthogonal reaction between DBCO-PEG and azide residues. In addition, XPS analysis revealed a significant decrease of the N1s signal, probably due to the shielding effect of PEG chains on the N-element-rich DOPA_4_-azide layer (Figure 2(d)). These results demonstrated the efficient PEGylation on the TiO_2_ surface via DOPA_4_-azide adhesion and DBCO-azide conjugation.

The antifouling property of the PEGylated surface was further examined by checking the inhibition of nonspecific cell adhesion. It is well known for vascular implants that excessive smooth muscle cell (SMC) growth and platelet adhesion will result in intimal hyperplasia and thrombosis [39], which are the main causes of device failure. As an example for illustration, here, we investigated the inhibitory effect of our PEGylated surfaces on SMCs and platelet adhesion. Human umbilical artery SMCs were first seeded on the TiO_2_, DOPA_4_-azide-coated, and PEGylated surfaces and cultured for 2, 24, and 72 h. The SMC adhesion and proliferation behaviors were investigated by fluorescence microscopy and CCK-8 (cell counting kit 8) assay. As shown in Figure 2(e), remarkable inhibition of SMC adhesion and growth on the PEGylated surface could be observed, and the inhibitory effect did persist for 3 days. In contrast, the original TiO_2_ surface and DOPA_4_-azide-coated surface showed strong SMC adhesion (nonspecific) and proliferation. Quantitative results by the CCK-8 assay and cell counting further confirmed the strong inhibitory effects of our PEGylated surface for SMC growth, in particular, the continuous inhibition of SMC adhesion (Figures 2(f) and 2(g)). In addition to SMCs, we also applied the PEGylated surface to test blood platelet adhesion. Likewise, bioorthogonal PEGylation on DOPA_4_-azide-coated TiO_2_ significantly inhibited the adhesion of platelets (Figures 2(h)–2(j)). Besides the significant reduction of platelet adhesion and spreading, the PEGylated surface also showed a low degree of fibrinogen adsorption and activation. Obviously, the bioorthogonal PEGylation assisted by mussel-inspired peptide adhesion would be a promising strategy for the fabrication of antifouling coatings on implanted biomaterials like stents, biosensors, and biochips.

### 2.4. Antibacterial Surface

Apart from the engineering of an antifouling surface, the bioinspired peptide mimic DOPA_4_-azide could also be used for the fabrication of an antimicrobial surface. For medical implants and devices (e.g., urinary catheters and orthopedic and dental implants), bacterial infections after implantation are associated with increased frequency and length of hospitalization as well as the risk of implant failure [40, 41]. Antibacterial functionalization of implants thus is highly demanded in the field of surface bioengineering. In this context, we designed a DBCO-modified antibacterial peptide (ABP), which was then used to engineer an antibacterial surface with the assistance of DOPA_4_-azide. Currently, there are only limited strategies for surface bioengineering of polymeric implants compared to metal implants, probably due to the chemical inertness of biomedically used polymer materials. Thus, we chose polyvinyl chloride (PVC) substrate (which is commonly used for medical tubes) to demonstrate the possibility of our method for biomodification of polymer implants.

**Figure 3:**
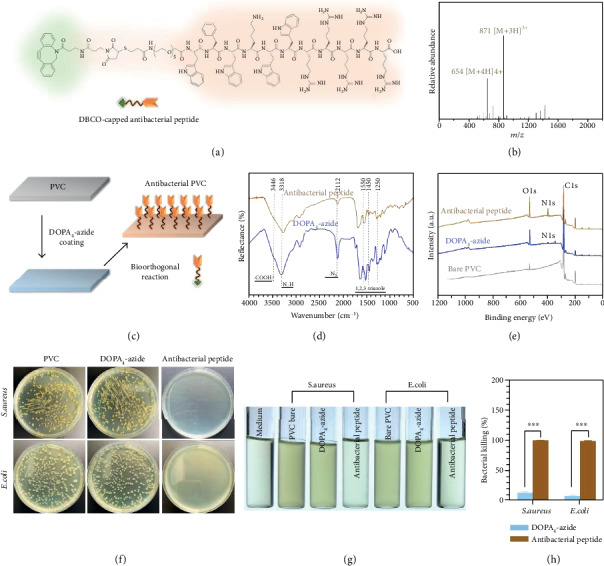
(a) Structural formula of the DBCO-modified antibacterial peptide (DBCO-ABP). (b) ESI mass spectrum of the DBCO-ABP. (c) Bioorthogonal conjugation of DBCO-ABP on DOPA_4_-azide-coated PVC substrates. (d) GATR-FTIR spectra of the DOPA_4_-azide-coated and ABP-modified surfaces. (e) XPS analysis of the PVC substrates after each step of surface treatments. (f) Agar plates observed after 24 h incubation of E. coli and S. aureus on the bare, DOPA_4_-azide-coated, and ABP-modified PVC substrates, respectively (plate sizes: 10 cm). (g) Photographs of the bacterial media after 12 h incubation with bare, DOPA_4_-azide-coated, and ABP-modified PVC substrates, respectively. (h) Quantitative analysis of bacterial killing efficiency by measuring the optical density at 600 nm based on the turbidity of the bacterial suspension. Statistically significant differences are indicated by ^∗∗∗^*p* < 0.001.

In this part, a representative ABP (HOOC-WFWKWWRRRRR-NH_2_) [42] was employed as the antibacterial backbone, which was linked with a PEG spacer and the DBCO group to obtain a DBCO-modified ABP (DBCO-ABP, Figure 3(a)). The ESI mass spectrum of the DBCO-ABP indicated the monoisotopic mass of [M+3H]^3+^ and [M+4H]^4+^ at 871 and 654 Da, respectively (Figure 3(b)). The result was in line with its theoretical molecular weight (2612.0 Da). The DBCO-ABP was then incubated with DOPA_4_-azide-coated PVC substrates to fabricate antibacterial surfaces (Figure 3(c)). As shown in Figure 3(d), there is a remarkable decrease of azide groups in the FTIR spectra, accompanied by the appearance of triazole after bioorthogonal conjugation. In addition, a significant increase of N1s signal and an appearance of S2p signal were found in the XPS spectrum of the ABP-modified surface (Figure 3(e) and Figure S2), due to the N-element-rich chemical composite of the DBCO-ABP layer. These results jointly confirmed the successful fabrication of ABP-modified PVC substrates by the combined use of DOPA_4_-azide and DBCO-ABP.

The antibacterial properties of ABP-modified PVC were further examined by using *E. coli* and *S. aureus*. A drop of bacterial suspension was distributed on the bare, DOPA_4_-azide-coated, and ABP-modified PVC substrates for solid culture tests, or the samples were fully immersed in bacterium suspensions for liquid culture. The ABP-modified surface showed potent bacterial inhibition in both solid and liquid media. As shown in Figure 3(f), no bacterial colony, regardless of Gram-positive or Gram-negative strains, was found on the ABP-modified surfaces after 24 h of culture. In contrast, the two control groups, including the bare and DOPA_4_-azide-coated surfaces, were both covered with a high density of bacterial colonies. A similar result was also observed in liquid media. The bacterial solutions incubated with ABP-modified PVC show a clear state, while the others appeared distinctly turbid in the bacterial suspension, implying the efficient inhibition of bacterial growth. According to the optical density at 600 nm (OD_600_), we found that more than 99% of the bacteria could be killed by the ABP layer in 12 h (Figures 3(g) and 3(h)). In addition, such potent inhibitory effect on bacteria could last for 1 month and more (Figure S3), indicating the durable antibacterial activity and also the high stability of the ABP layer. The above study confirmed the successful fabrication of an antibacterial surface, indicating the high applicability of our bioclickable mussel-inspired peptide mimic for surface engineering antibacterial coatings on the medically used, in particular, polymer-based implants.

### 2.5. Antithrombogenic Surface

As one of the most common physiological and pathological phenomena, thrombosis occurs as a host defense mechanism to preserve the integrity of the closed circulatory system after vascular damages [43]. However, the development of clots in circulation after therapeutic intervention is the most frequent cause of morbidity and mortality. Particularly, in the field of cardiovascular stents, chronic and acute interfacial thrombogenesis inevitably happens due to the vascular injury caused by stent expansion [44]. Thus, biofunctionalized stents with antithrombotic activity are highly desired. In this context, we further demonstrated the potential of our methods for the fabrication of an antithrombotic surface.

**Figure 4:**
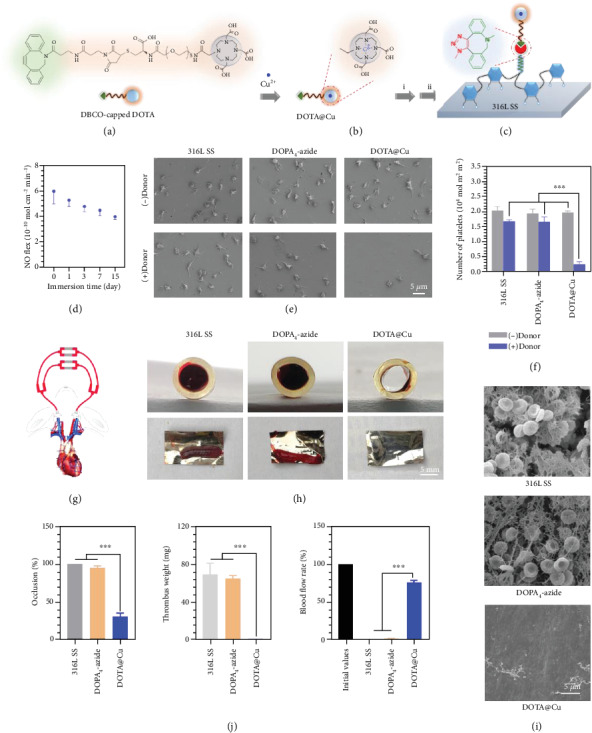
(a) Structural formula of the DBCO-modified cyclen DBCO-DOTA with the ability to chelate Cu(II). (b, c) Cu(II) chelation and bioorthogonal conjugation to form a DOTA@Cu-modified 316L SS substrate. (d) Time-dependent NO generation from the DOTA@Cu-modified 316L SS substrate. (e, f) SEM images and numbers of adherent platelets after incubation with different 316L SS substrates. (g) Schematic illustration of the rabbit AV shunt model. (h) Cross-sectional photographs of tubing and the corresponding thrombus in different groups. (i) SEM images of platelet activation and fibrinogen activation on different 316L SS substrates. (j) Quantitative results of the thrombus weight, blood flow, and occlusion rate in different groups. Statistically significant differences are indicated by ^∗∗∗^*p* < 0.001.

The NO (nitric oxide, a gaseous signaling molecule)-generating compounds have been well studied for surface engineering of vascular stents to prevent platelet activation and aggregation, inhibit thrombogenesis, suppress SMC proliferation, promote EC growth, etc. [45, 46]. Accordingly, we designed a DBCO-capped NO-generating compound for surface engineering. As is well studied in previous work, the transition metal ion Cu(II) has excellent glutathione peroxidase- (GPx-) like activity [47, 48], which can catalytically generate NO from both endogenous and synthetic S-nitrosothiols (RSNOs) by decomposing them in the presence of reduced glutathione (GSH). In order to immobilize Cu ions, a cyclen DOTA (1,4,7,10-tetraazacyclododecane-*N*,*N*′,*N*^″^,*N*^‴^-tetraacetic acid) was conjugated with a DBCO group (Figure 4(a) and Figure S4) [49]. The Cu(II)-cyclen complex (DOTA@Cu) thus could be bioorthogonally conjugated on a DOPA_4_-azide-coated substrate to obtain a NO-generating surface (Figures 4(b) and 4(c)). In this study, 316L stainless steel (SS) foil was used as the model substrate since the material is widely used for vascular stents.

After DOTA@Cu modification (Figure S5), the *in vitro* NO-releasing property was first determined by a real-time chemiluminescent assay. PBS solution containing 10 *μ*M reducing agent GSH and 10 *μΜ* S-nitrosoglutathione (GSNO, an endogenous NO donor) [50] was used to simulate the blood environment. Real-time monitoring of the NO flux revealed a steady NO generation from the DOTA@Cu-modified surface (Figure S6). Ageing studies showed that efficient NO release could last for more than 2 weeks (Figure 4(d)), indicating the suitability for long-term use.

Since thrombogenesis involves a series of biochemical processes like platelet aggregation, coagulation, and fibrinolysis [43], we then checked the *in vitro* antiplatelet property. Without donor supply, all surfaces induced substantial platelet adhesion and activation in 30 min, and the DOTA@Cu-modified 316L SS foil showed almost no inhibition in the amount and activation rates of adherent platelets (Figures 4(e) and 4(f)). Upon the addition of the NO donor, significant changes were observed on the DOTA@Cu-modified surface. The controls (i.e., the bare and DOPA_4_-azide-coated 316L SS substrates) had evident platelet adhesion, and the spread morphology of platelets indicated a high degree of activation and aggregation. In contrast, the DOTA@Cu-modified 316L SS foil showed substantially reduced platelet adhesions with an inactive spherical state. With the positive result *in vitro*, we then investigated the antithrombogenic property using *ex vivo* perfusion experiments. The control and DOTA@Cu-modified 316L SS foils were curled up and placed onto the inner walls of commercially available cardiopulmonary perfusion tubes, which were then connected to a rabbit arteriovenous (AV) shunt (Figure 4(g)) [45]. The ability of different groups to support blood flow was evaluated in the presence of the NO donor. After 2 h of *ex vivo* circulation, the sizes of occlusive thrombosis, thrombus weight, and blood flow rates in the circuit were evaluated (Figures 4(h)–4(j)). Optical microscope photos and SEM images both showed serious thrombus formation on the two control groups (i.e., the bare and DOPA_4_-azide-coated 316L SS foils). In contrast, only a small number of cruor were observed on the NO-releasing DOTA@Cu-modified foil. These results jointly confirmed the perfect hemocompatibility and antithrombogenic property of the DOTA@Cu-modified surface. It can be concluded that this study demonstrated the potential of our clickable peptide mimic for surface bioengineering of vascular implants with high antithrombogenic activity.

## 3. Conclusion

In summary, we upgraded current mussel-inspired surface engineering strategies by the combination of mussel adhesive peptide mimicking and bioorthogonal click chemistry. The mainline of this work is a novel mussel adhesive peptide mimic capped with a bioclickable azide group (i.e., DOPA_4_-azide). Similar to the mussel adhesion mechanism, the peptide mimic DOPA_4_-azide could stably bind onto a broad range of materials via covalent/noncovalent comediated molecular adhesion. In addition, the azide residues on the DOPA_4_-azide-bound surfaces enabled a second-step specific grafting of DBCO-modified bioactive ligands through click reaction. To demonstrate the applicability of our strategy for diversified biofunctionalization, we bioorthogonally conjugated three typical biomolecules on different substrates. The results verified the feasibility to fabricate functional surfaces that matched highly with some essential requirements of medical implants, for instance, the antifouling, antibacterial, and antithrombogenic activity. Overall, this novel surface bioengineering strategy has shown broad applicability in both the types of substrate materials and the intended bioactivities. The molecular modification of bioorthogonal chemistry without hazardous side products and the universality of mussel-inspired molecular adhesion synergistically provide a versatile surface bioengineering strategy for a wide range of biomedical materials.

## 4. Materials and Methods

### 4.1. DOPA_4_-Azide Synthesis and Coating

The bioclickable mussel-inspired peptide mimic (DOPA)_4_-azide was synthesized through Fmoc-mediated solid-phase synthesis according to a previously reported method [33–35]. (DOPA)_4_-azide and the DBCO-modified molecules (DBCO-ABP and DBCO-DOTA) were synthesized with the assistance of ChinaPeptides Co. Ltd. (Shanghai, China, purity > 95%). DBCO-PEG (Mw = 5000) was purchased from Nanocs Inc. (New York, NY, USA). Phosphate-buffered saline solution (PBS, 0.02 mM, pH 7.2) was prepared in ultrapurified water (purified with Thermo Scientific Barnstead NANOpure Diamond Water Purification Systems to give a minimum resistivity of 18.2 M*Ω*·cm) and a purchased phosphate buffer salt (Beyotime Biotechnology, China). All substrates (Au, Cu, Ti, Si, TiNi, 316L SS, TiO_2_, Glass, PVC, PTFE, PET, PU, and PS) were first washed by ultrapure water, ethanol, and hydrogen peroxide/ammonia (1 : 1) three times, respectively, then dried in a stream of dry nitrogen. Then, they were immersed in a PBS of DOPA_4_-azide (0.1 mg·mL^−1^) for 24 h at room temperature, washed by ultrapure water three times, and dried with a stream of dry nitrogen.

### 4.2. Bioorthogonal Grafting

Different substrates with DOPA_4_-azide coating (DBCO-PEG for TiO_2_ deposited quartz substrate, DBCO-ABP for PVC, and DBCO-DOTA for 316L SS) were dipped into PBS solutions containing 1 mg·mL^−1^ DBCO-modified molecules for 24 h in room temperature; then, the substrates were washed by ultrapure water three times and dried with a stream of dry nitrogen. For the bioorthogonal grafting process of DBCO-DOTA, an additional CuCl_2_·2H_2_O solution (0.014 mg·mL^−1^) was used in order to get a grafted surface with copper(II)-chelated DOTA (DOTA@Cu(II)). In the graphs above, the materials without coating were named 316L SS, PVC, and TiO_2_, respectively; the samples with DOPA-azide coating were named as DOPA_4_-azide; TiO_2_ surface with PEG coating was named as PEG; PVC with ABP coating was named as antibacterial peptide; and 316L SS with DOTA@Cu(II) coating was named as DOTA@Cu.

### 4.3. Characterization

A grazing incidence attenuated total reflection Fourier transform infrared (GATR-FTIR) spectrum was taken with a Nicolet model 5700 instrument. X-ray photoelectron spectroscopy (XPS) (K-alpha, ThermoFisher, USA), with an excitation source monochromatic Al K*α* (1486.6 eV), was used to detect the surface elemental compositions. The nuclear magnetic resonance (NMR) spectrum (Bruker AVANCE III 400) was used to analyze DOPA_4_-azide (DMSO-d6). The scanning electron microscope (SEM) (ZEISS EVO 18) was used to characterize the morphology and the number of the platelets. Electron Paramagnetic Resonance (EPR) spectra were measured on a Bruker EPR EMXPlus with 5 mg sample (X-band is 9.85 GHz, field modulation is 100 kHz, and the power is 0.2 mW). The NO generating property was measured by using a chemiluminescence NO analyzer (NOA) (Sievers 280i, Boulder, CO). Laser scanning confocal microscopy (LSCM, A1 Plus, Nikon, Japan) was used for cell morphology observation.

### 4.4. Cell Adhesion and Proliferation

In order to evaluate the antibiofouling ability, human umbilical artery smooth muscle cells (HUASMCs), with a density of 5 × 10^4^ cells·mL^−1^, were seeded on the samples and cultured for 2, 24, and 72 h, The proliferation efficiencies of HUASMCs were evaluated by cell counting and CCK-8 assay at 24 and 72 h.

### 4.5. Platelet Adhesion

Fresh human whole blood was legally obtained from the central blood station of Chengdu, China, following ethical standards. Platelet-rich plasma (PRP) was obtained by centrifuging fresh human whole blood at 1500 rpm for 15 min. To assess the antiplatelet performance of DOTA@Cu-modified 316L SS, 120 *μ*L of PRP was added and incubated at 37°C for 20 min, followed by rinsing with saline solution three times. The samples were divided into two groups, with or without NO donor (10 *μΜ* GSNO and 10 *μΜ* GSH) supplement.

### 4.6. Antibacterial Activity in Solid Medium

The antibacterial properties were investigated by using *E. coli* and *S. aureus.* Bacteria were precultured in solid medium (yeast extract 5 g·L^−1^, tryptone 10 g·L^−1^, NaCl 10 g·L^−1^, and agar 15 g·L^−1^, dissolved with ultrapure water) for 24 h at 37°C and subcultured twice to make it a monoclonal bacterium. Fresh bacterial colonies (1-2 rings) on the solid medium, picked with the inoculating loop, were dissolved with the 0.2% liquid medium/99.8% NaCl (liquid medium: yeast extract 5 g·L^−1^, tryptone 10 g·L^−1^, and NaCl 10 g·L^−1^, dissolved with ultrapure water). Tenfold increasing sequential dilutions were made, and the concentration was adjusted to 5.0 × l0^5^ ~ 106 CFU mL^−1^. 200 *μ*L of the above bacterial solution was separately added on the surface of samples and covered with a film to keep it wet. All samples were placed in an incubator at 37°C for 24 h. After that, the surface bacteria were rinsed and dissolved in saline solution (20 mL). 200 *μ*L of the above bacterial solution was added to the solid medium and cultured for 24 h at 37°C. Finally, the colonies on the solid medium were counted.

### 4.7. Antibacterial Activity in Solution

After preculture in solid medium, fresh bacterial colonies (1-2 rings) were transferred to liquid medium and cultivated for 24 h then diluted for 10^6^ times with liquid medium. All samples were placed in a 24-well plate. 200 *μ*L diluted bacterial solution (10^6^ CFU·mL^−1^) was added at 37°C. After 12 h, 800 *μ*L liquid medium was added to the samples and cultivated for 12 h. Finally, 200 *μ*L solution was taken out, and the OD value at 600 nm was measured.

### 4.8. Fibrinogen Adhesion and Activation

Fresh human whole blood was acquired from the central blood station of Chengdu, China, following all the ethical standards. The blood and trisodium citrate were mixed in a 9 : 1 volumetric ratio. Platelet poor plasma (PPP) was obtained by centrifuging (3000 rpm, 15 min) the mixture. 100 *μ*L of PPP was distributed on the substrates (10 mm × 10 mm) and incubated for 2 h in a 37° C water bath, followed by PBS washing for 3 times. 300 *μ*L of blocking solution (5 g of bovine serum albumin dissolved in 100 mL of 0.9% NaCl solution) was added on the substrates for 30 min at 37°C, followed by PBS washing for 3 times. 50 *μ*L HRP-labeled mouse anti-human fibrinogen antibody was further added and incubated for 1 h at 37°C. After washing with PBS, they were reacted with 100 *μ*L of tetramethylbenzidine (TMB) chromogenic solution for 10 min. Finally, 50 *μ*L of 1 M H_2_SO_4_ was added to stop the color reaction, and a microplate reader was used to examine the optical density. The test of fibrinogen activation is using anti-fibrinogen gamma chain mouse monoclonal antibody as primary antibody and HRP-labeled goat anti-mouse antibody as secondary antibody; the detailed detection was the same as described above.

### 4.9. *Ex Vivo* Thrombogenicity

All the experiments on animals obeyed the Local Ethical Committee and Laboratory Animal Administration Rules of China. The DOTA@Cu-coated 316L SS foils (0.2 mm × 9 mm × 15 mm) were rolled into a bucket and inserted into the PVC catheters. After injection of pentobarbital sodium (30 mg/mL, 1 mL per kg) from the ear, the left carotid artery and right external jugular vein of three adult New Zealand white rabbits (2.2–2.5 kg) were exposed. Then, the PVC catheter was connected to the blood vessel to form a closed loop. The samples were taken out after a two-hour cycle and rinsed with saline solution three times. The cross-sections of the catheters were photographed to calculate the occlusive rates. The weight of the thrombus formed was also weighed and analyzed by SEM after fixation by glutaraldehyde (2.5%).

### 4.10. Statistical Analysis

All data in this study are exhibited as the mean ± standard deviation. Statistical analysis was conducted by applying SPSS software, employing a one-way ANOVA as detailed in figure captions. Tests that have an alpha level for significance set at *p* < 0.05 were considered significantly different. All of the tests were performed at least three times with no less than four parallel samples.

## Data Availability

The data that support the findings of this study are available from the corresponding authors on request.

## Supplementary Materials

Supplementary Materials Figure S1: XPS spectra of different substrates (Au, Cu, Ti, Si, TiNi, 316L SS, Glass, PVC, PET, PU, and PS) after DOPA_4_-azide coating. Figure S2: XPS spectra of the DOPA_4_-azide-coated surface before and after ABP grafting, and the high-resolution S2p XPS spectra of the ABP coating. The presence of elemental sulfur indicates successful grafting of ABP on the DOPA_4_-azide coating. Figure S3: stability of antimicrobial properties of ABP coating. The ABP coating was soaked in PBS buffer and retained antimicrobial rate of 90 percent for 7 days and 80 percent for 15 days. Figure S4: structural formula and mass spectrometry of the DBCO-DOTA molecule; the 607.2[M+2H]^2+^ and 1213[M+H]^+^ indicate that the relative molecular weight of the synthetic DBCO-DOTA molecule is around 1212, basically the same as the structural formula. Figure S5: (A) XPS spectra of the DOPA_4_-azide coating before and after DOTA@Cu grafting, and high-resolution Cu2p XPS spectra of the DOTA@Cu coating. Figure S6: (A) GATR-FTIR spectra of DOPA_4_-azide coating before and after DOTA@Cu grafting, the disappearance of -N3 stretching, and the appearance of 1,2,3-triazole, respectively, indicate the successful grafting of DBCO-DOTA@Cu to the DOPA_4_-azide coating via click reaction. (B) Catalytic NO generation patterns induced by DOTA@Cu coatings in deoxygenated PBS (pH 7.4) containing 10 *μ*M GSNO and 10 *μ*M GSH at 37°C.
