# Corrigendum to “A Versatile Surface Bioengineering Strategy Based on Mussel-Inspired and Bioclickable Peptide Mimic”

**DOI:** 10.34133/2021/9864698

**Published:** 2021-06-30

**Authors:** Yu Xiao, Wenxuan Wang, Xiaohua Tian, Xing Tan, Tong Yang, Peng Gao, Kaiqing Xiong, Qiufen Tu, Miao Wang, Manfred F. Maitz, Nan Huang, Guoqing Pan, Zhilu Yang

**Affiliations:** ^1^Key Laboratory of Advanced Technologies of Materials, Ministry of Education, School of Materials Science and Engineering, Southwest Jiaotong University, Chengdu, Sichuan 610031, China; ^2^Institute for Advanced Materials, School of Materials Science and Engineering, Jiangsu University, Zhenjiang, Jiangsu 212013, China; ^3^Max Bergmann Center of Biomaterials, Leibniz Institute of Polymer Research Dresden, Hohe Strasse 6, 01069 Dresden, Germany

In the article titled, “A Versatile Surface Bioengineering Strategy Based on Mussel-Inspired and Bioclickable Peptide Mimic” [1], there was an error in Figure 2. In panel (e), the cell pictures of PEG after culture for 24 and 72 h were updated. The corrected figure is shown and is listed as Figure 1.

## Figures and Tables

**Figure 1:**
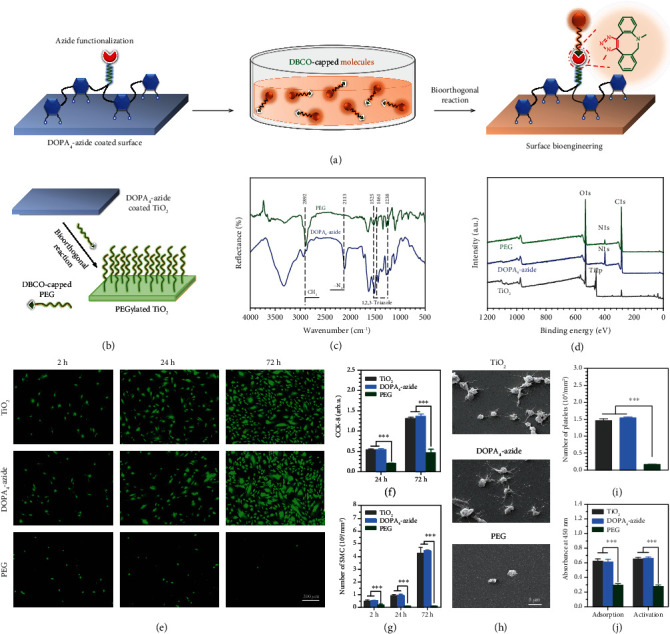
(a) Schematic illustration of the DOPA_4_-azide coated substrate for second-step surface biomodification through bioorthogonal DBCO-azide click reaction. (b) Bioorthogonal PEGylation on the TiO_2_ surface using DBCO-PEG. (c) GATR-FTIR spectra of the DOPA_4_-azide coated and PEGylated surfaces. (d) XPS analysis of the TiO_2_ surfaces at each step of surface treatments. (e) SMC adhesion at 2, 24, and 72 h. (f, g) SMC proliferation by the CCK-8 assay and cell counting. (h) Scanning electron microscope (SEM) images of adherent blood platelets. (i) Average numbers of adherent blood platelets. (j) Fibrinogen absorption and activation. Statistically significant differences are indicated by *p* < 0.001.
