# Supplementary material for: A Versatile Surface Bioengineering Strategy Based on Mussel-Inspired and Bioclickable Peptide Mimic
Source: Research (Wash D C). 2020 Jun 25;2020:7236946. doi: 10.34133/2020/7236946 (PMC7334800; doi:10.34133/2020/7236946)
Supplement: Supplementary Materials — Figure S1: XPS spectra of different substrates (Au, Cu, Ti, Si, TiNi, 316L SS, Glass, PVC, PET, PU, and PS) after DOPA4-azide coating. Figure S2: XPS spectra of the DOPA4-azide-coated surface before and after ABP grafting, and the high-resolution S2p XPS spectra of the ABP coating. The presence of elemental sulfur indicates successful grafting of ABP on the DOPA4-azide coating. Figure S3: stability of antimicrobial properties of ABP coating. The ABP coating was soaked in PBS buffer and retained antimicrobial rate of 90 percent for 7 days and 80 percent for 15 days. Figure S4: structural formula and mass spectrometry of the DBCO-DOTA molecule; the 607.2[M+2H]2+ and 1213[M+H]+ indicate that the relative molecular weight of the synthetic DBCO-DOTA molecule is around 1212, basically the same as the structural formula. Figure S5: (A) XPS spectra of the DOPA4-azide coating before and after DOTA@Cu grafting, and high-resolution Cu2p XPS spectra of the DOTA@Cu coating. Figure S6: (A) GATR-FTIR spectra of DOPA4-azide coating before and after DOTA@Cu grafting, the disappearance of -N3 stretching, and the appearance of 1,2,3-triazole, respectively, indicate the successful grafting of DBCO-DOTA@Cu to the DOPA4-azide coating via click reaction. (B) Catalytic NO generation patterns induced by DOTA@Cu coatings in deoxygenated PBS (pH 7.4) containing 10 μM GSNO and 10 μM GSH at 37°C. [file 7236946.f1.docx]

*Supplemental Material*

**A Versatile Surface Bioengineering Strategy Based on Mussel-Inspired and Bioclickable Peptide Mimic**

Yu Xiao,^1^ Wenxuan Wang,^1^ Xiaohua Tian,^2^ Xing Tan,^1^ Tong Yang,^1^ Peng Gao,^1^ Kaiqing Xiong,^1^ Qiufen Tu,^1^ Miao Wang,^2^ Manfred F. Maitz,^1,3^ Nan Huang,^1^ Guoqing Pan^2,^* and Zhilu Yang^1,^*

^1^ Key Laboratory of Advanced Technologies of Materials, Ministry of Education, School of Materials Science and Engineering, Southwest Jiaotong University, Chengdu, Sichuan 610031, China.

^2^ Institute for Advanced Materials, School of Materials Science and Engineering, Jiangsu University, Zhenjiang, Jiangsu 212013, China.

^3^ Max Bergmann Center of Biomaterials, Leibniz Institute of Polymer Research Dresden, Hohe Strasse 6, 01069 Dresden, Germany

* Correspondence should be addressed to Guoqing Pan; panguoqing@ujs.edu.cn and Zhilu Yang; zhiluyang1029@swjtu.edu.cn


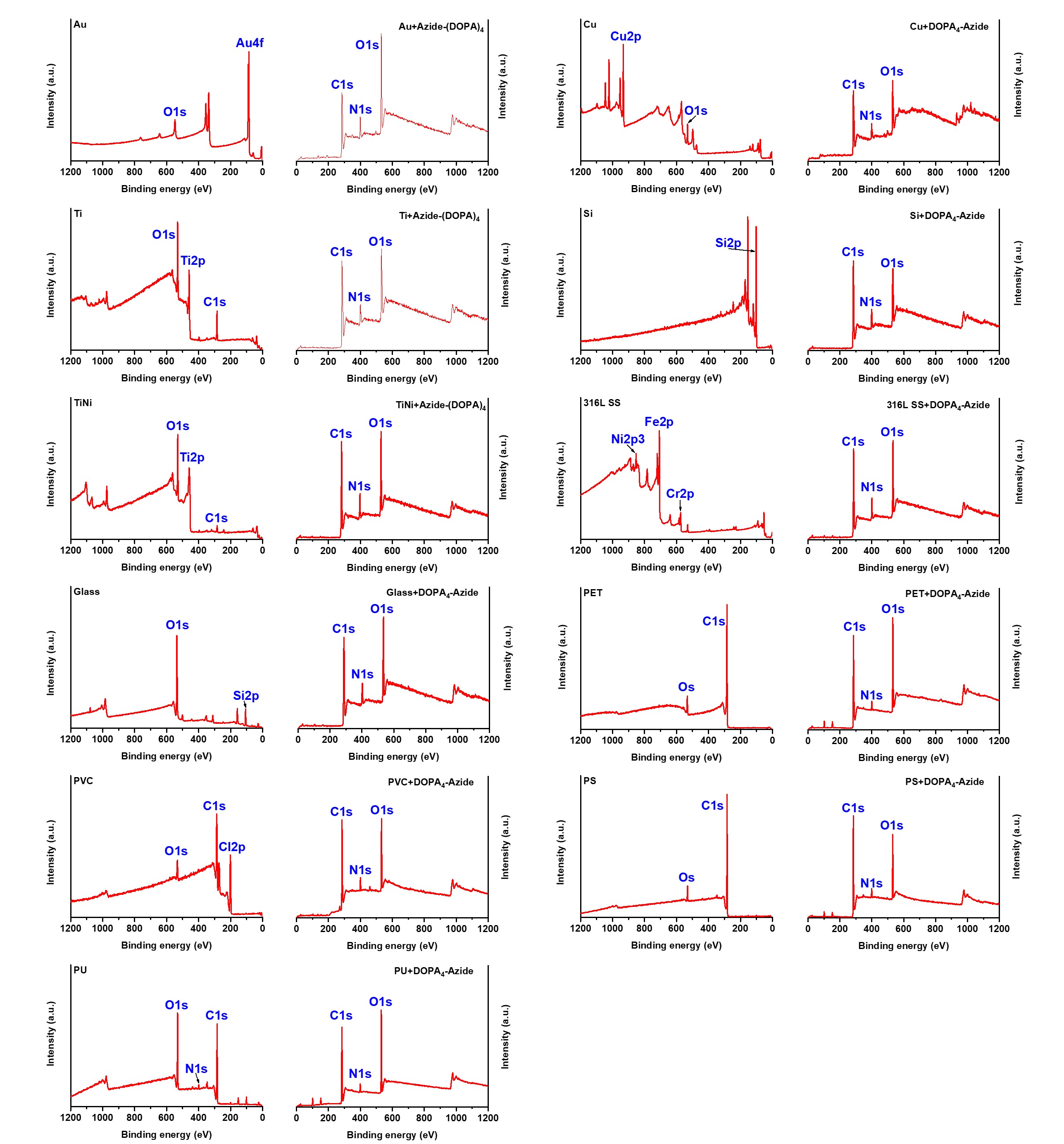


**Figure S1.** XPS spectra of different substrates (Au, Cu, Ti, Si, TiNi, 316L SS, Glass, PVC, PET, PU, PS) after DOPA_4_-Azide coating.


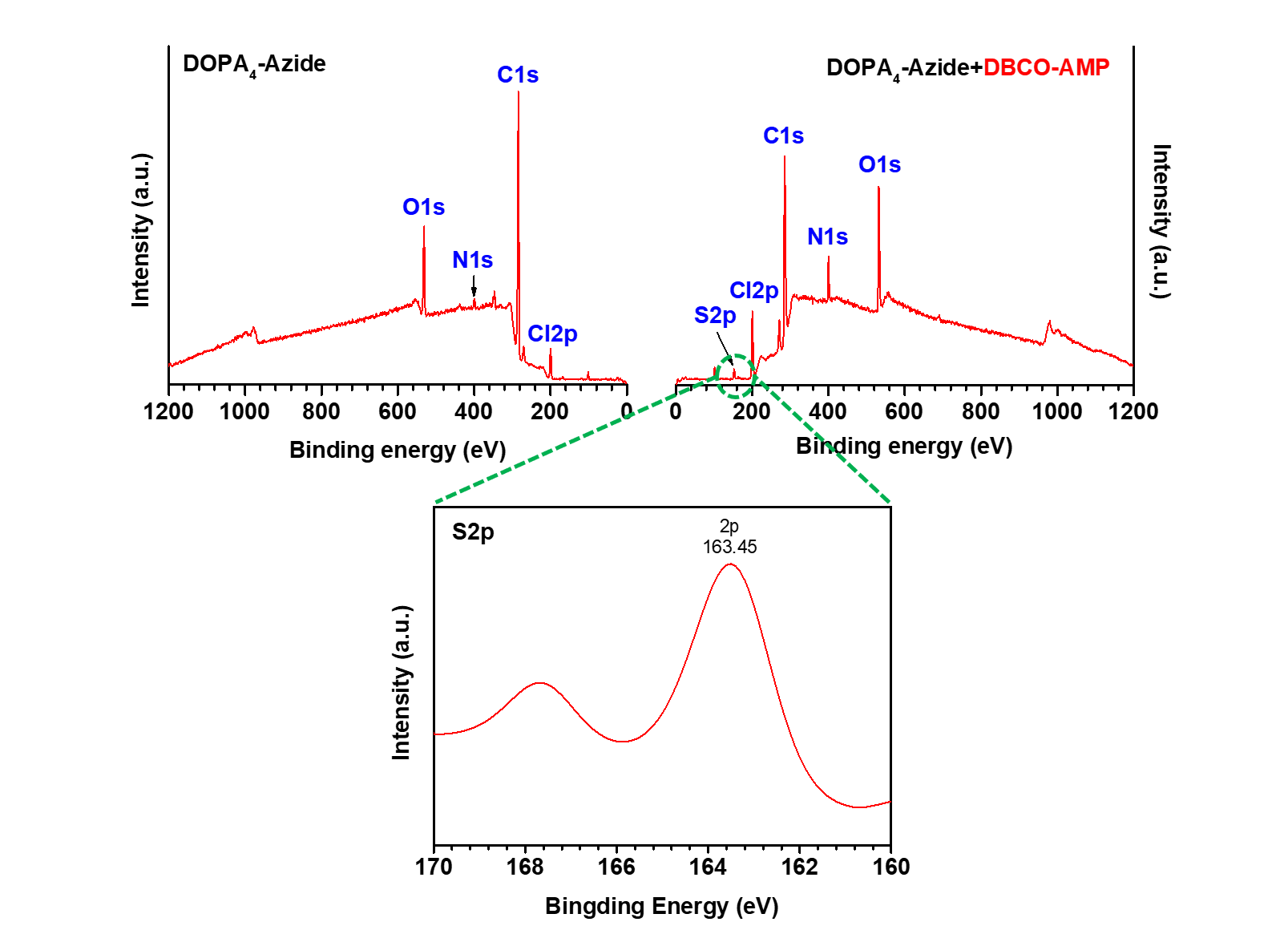


**Figure S2.** XPS spectra of the DOPA_4_-Azide-coated surface before and after ABP grafting, and the high-resolution S2p XPS spectra of the ABP coating. The presence of elemental sulfur indicates successful grafting of ABP on the DOPA_4_-Azide coating.


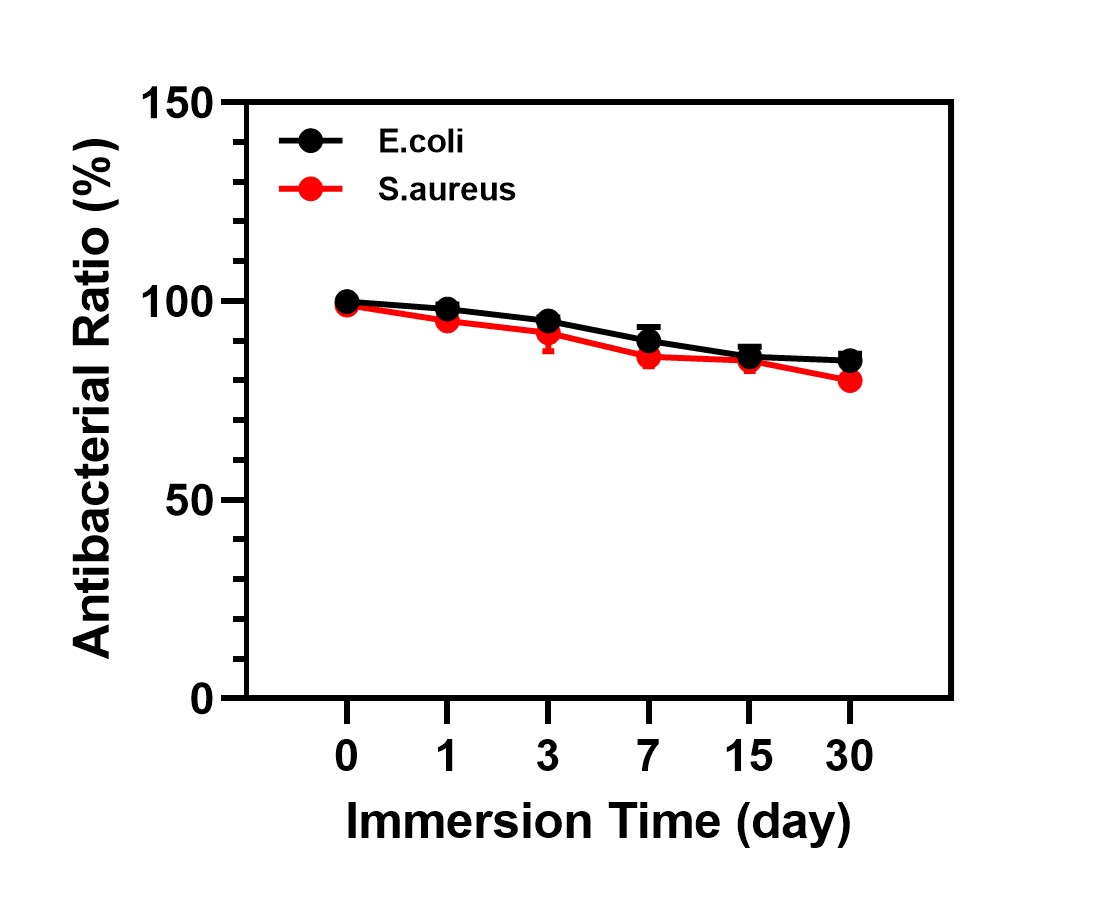


**Figure S3.** Stability of antimicrobial properties of ABP coating. The ABP coating was soaked in PBS buffer and retained antimicrobial rate of 90 percent for 7 days and 80 percent for 15 days.


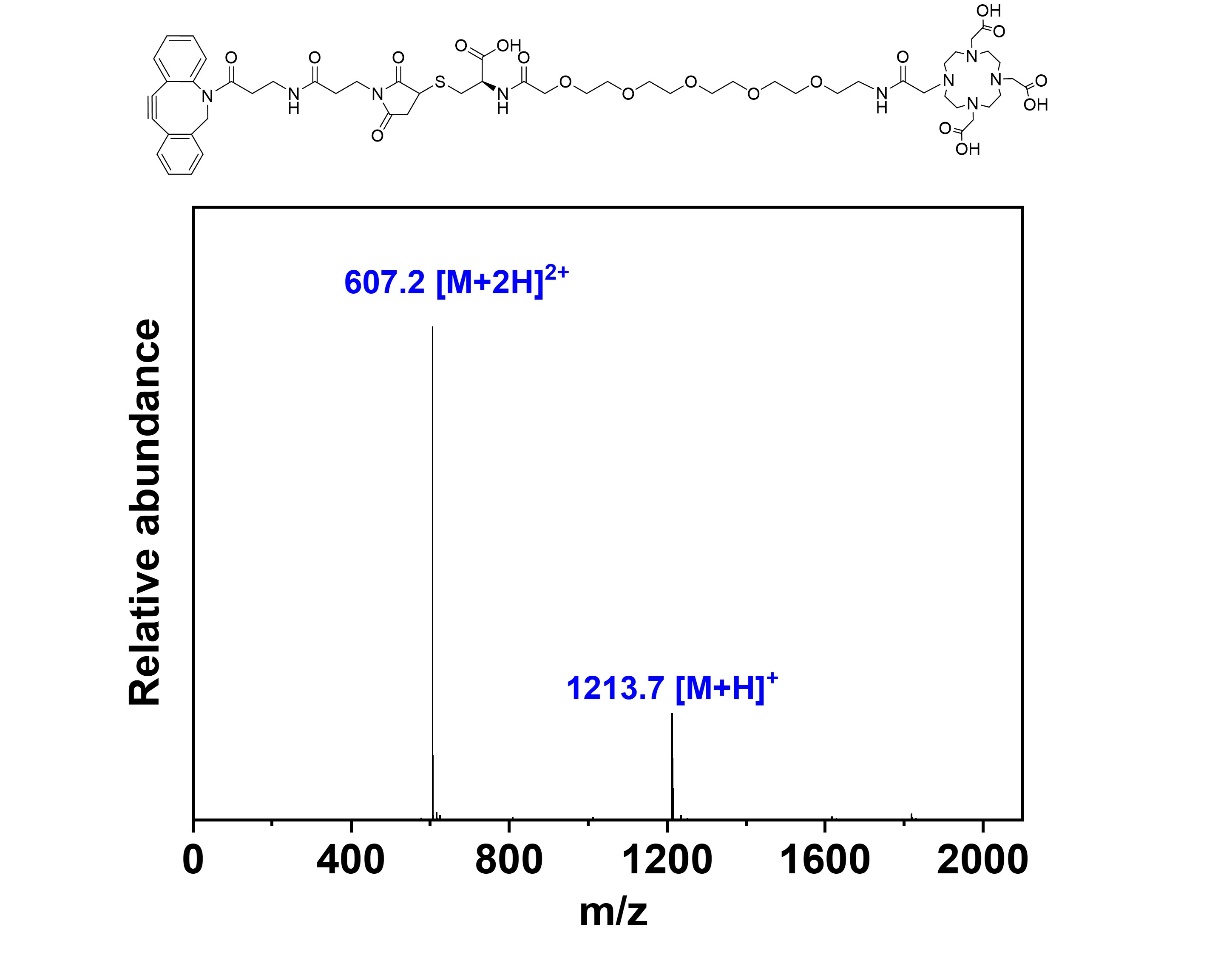


**Figure S4.** Structural formula and mass spectrometry of the DBCO-DOTA molecule, the 607.2[M+2H]^2+^ and 1213[M+H]^+^ indicate the relative molecular weight of the synthetic DBCO-DOTA molecule is around 1212, basically the same with the structural formula.


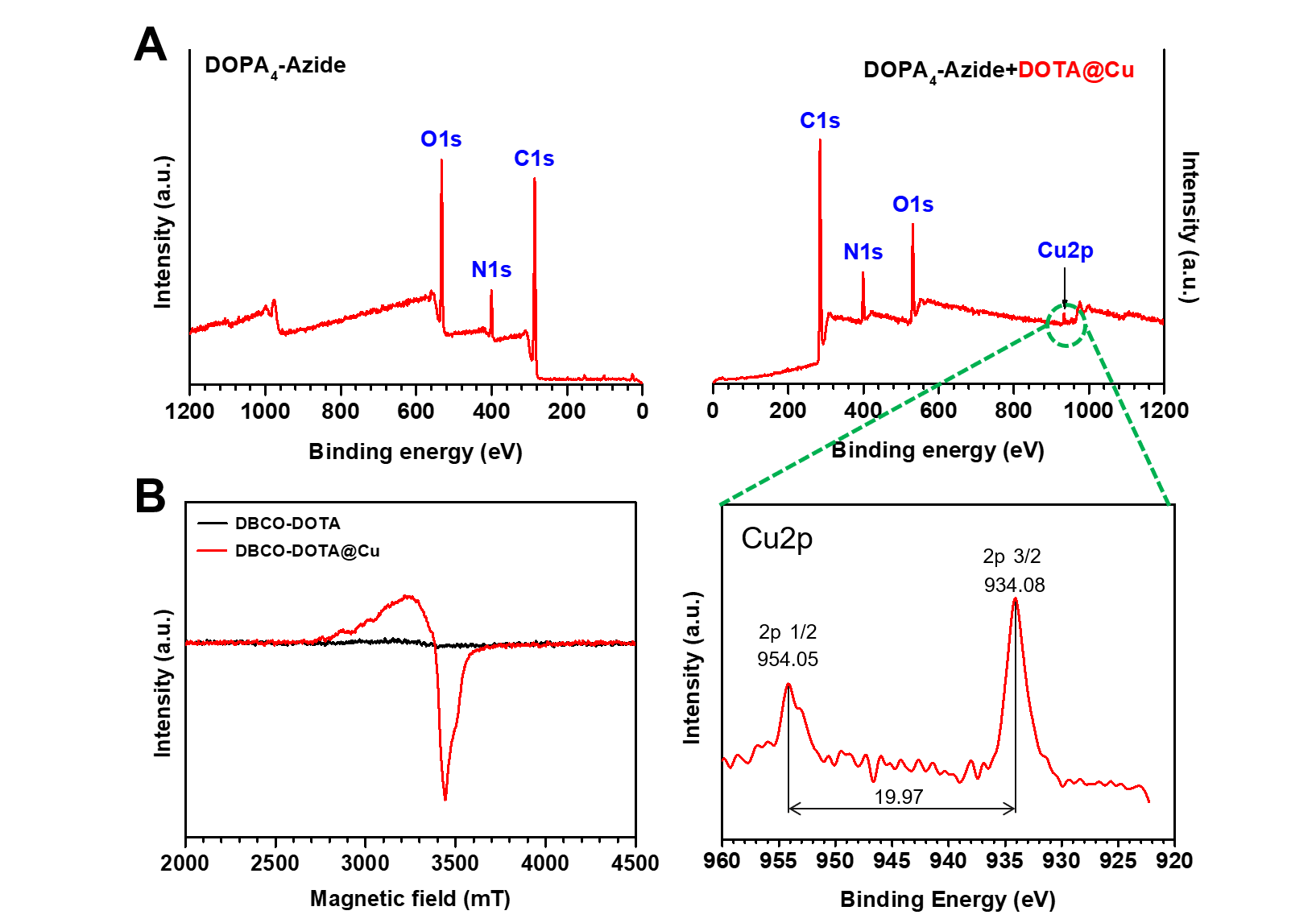


**Figure S5.** (A) XPS spectra of the DOPA_4_-Azide coating before and after DOTA@Cu grafting, and high-resolution Cu2p XPS spectra of the DOTA@Cu coating. The presence of elemental copper indicates successful grafting of DOTA@Cu on the DOPA_4_-Azide coating. (B) EPR of DBCO-DOTA and DBCO-DOTA@Cu, EPR spectra revealed the presence of Cu^II^-DOTA signal in 3400-3500 mT, indicating the possible formation of Cu^II^-DOTA coordination complexes.


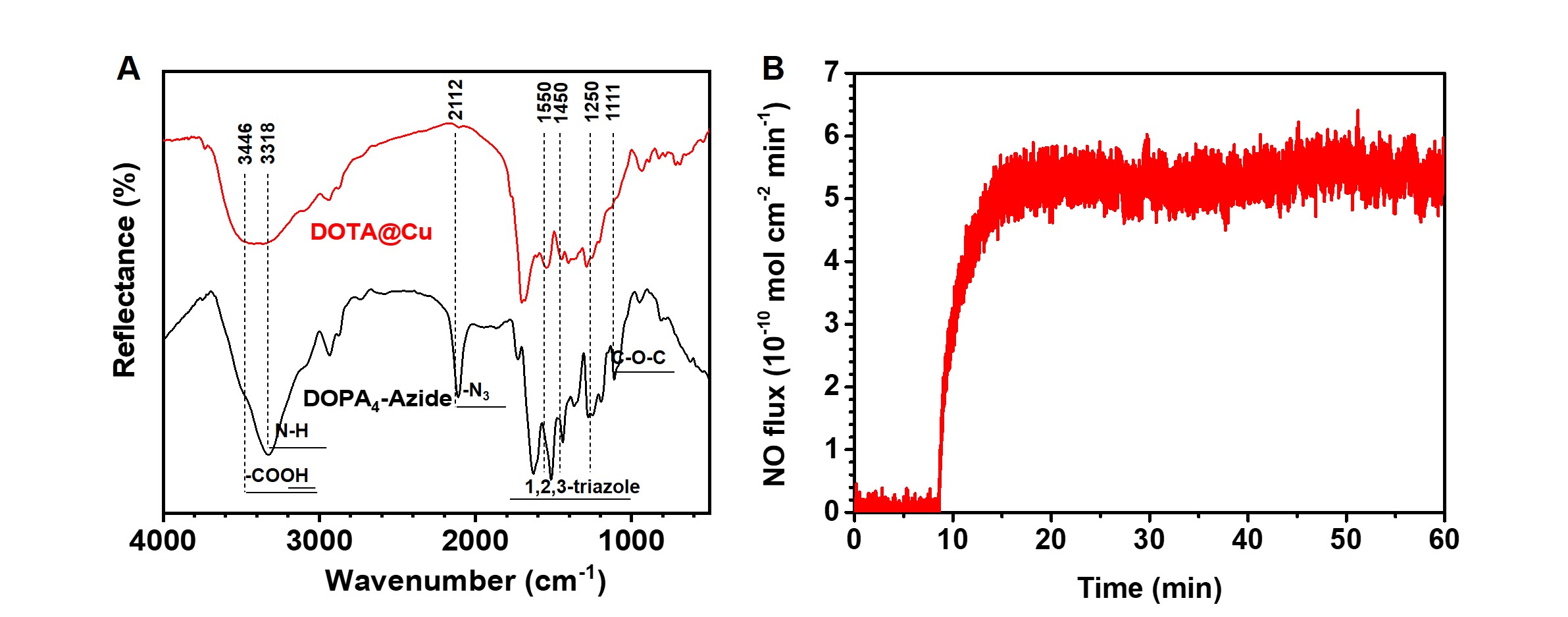


**Figure S6.** (A) GATR-FTIR spectra of DOPA_4_-Azide coating before and after DOTA@Cu grafting, the disappearance of -N_3_ stretching, and the appearance of 1,2,3-triazole, respectively, indicate the successful grafting of DBCO-DOTA@Cu to the DOPA_4_-Azide coating via click reaction. (B) Catalytic NO generation patterns induced by DOTA@Cu coatings in deoxygenated PBS (pH 7.4) containing 10 μM GSNO and 10 μM GSH at 37 °C.
